# VSELs and OSCs together sustain oogenesis in adult ovaries and their dysfunction results in age-related senescence, PCOS, POI and cancer

**DOI:** 10.1186/s13048-022-01093-y

**Published:** 2023-02-01

**Authors:** Deepa Bhartiya, Diksha Sharma

**Affiliations:** 1 Stem Cell Biology Department, ICMR-National Institute for Research in Reproductive & Child Health, Jehangir Merwanji Street, Parel, Mumbai 400 012 India; 2 Present address: Epigeneres Biotech Pvt Ltd, Sun Mill Compound, Senapati Bapat Marg, Lower Parel, Mumbai, 400 013 India

**Keywords:** Ovary, Stem cells, Ovary surface epithelium, Very small embryonic-like stem cells (VSELs), Ovarian stem cells (OSCs), Germ cell nests

## Abstract

Multiple studies using single-cell RNA sequencing (scRNAseq) have failed to detect stem cells in adult ovaries. We have maintained that two populations of ovarian stem cells including pluripotent, very small embryonic-like stem cells (VSELs) and tissue-committed ‘progenitors’ termed ovarian stem cells (OSCs) can easily be detected in Hematoxylin and Eosin-stained ovary surface epithelial (OSE) cells smears prepared from both mice and human ovaries. Most likely the stem cells never get subjected to scRNAseq since they pellet down only by centrifuging cells suspension at 1000 g while cells for scRNAseq were invariably prepared by centrifuging at 200-400 g. A recent article provided further explanation for the failure of scRNAseq to detect ovarian stem cells. Extensive reanalysis of data (generated by scRNAseq) using an advanced software successfully detected OSCs and meiotic markers supporting neo-oogenesis in adult human ovaries. But this article remained critical on the biological relevance of VSELs and their relationship with OSCs. By carefully studying the OSE cells smears (which hold VSELs, OSCs and germ cell nests GCNs), prepared by partial trypsin digestion of intact mice ovaries during different stages of estrus cycle, we have successfully delineated novel functions of VSELs/OSCs in vivo under physiological conditions. VSELs undergo asymmetrical divisions to self-renew and give rise to slightly bigger OSCs which in turn undergo symmetrical divisions and clonal expansion to form GCNs, regular neo-oogenesis and follicle assembly. GCNs have been earlier described in fetal ovaries and during OSE cells culture (from adult ovaries) in response to FSH treatment. Dysfunction of VSELs/OSCs (which express ERα, ERβ, FSHR) due to neonatal exposure to endocrine disruption results in ovarian insufficiency and polycystic ovaries. VSELs have also been implicated in ovarian cancer. Age-related ovarian senescence/menopause is also due to dysfunction and blocked differentiation of VSELs/OSCs. These novel findings in vivo along with abundant in vitro and lineage tracing studies data in published literature provides huge scope for further research, offers novel avenues to manage ovarian pathologies and calls for re-writing of textbooks.

## Main text

Single-cell RNAseq is an important and powerful advance as it provides meaningful information on individual cells, but the data is often noisy and complicated, making computational analysis challenging. This was well exemplified by scRNAseq study reported by Wagner et al. [1] which erroneously concluded that there are no oogonial stem cells in adult ovaries. This attracted a commentary [2] and recent article published in the journal Stem Cells provided additional explanation why Wagner’s group failed to detect OSCs [3]. Extensive reanalysis of data using an advanced software successfully detected OSCs and active meiotic divisions suggesting neo-oogenesis in adult ovaries. Also, the perivascular cells were wrongly isolated as ‘OSCs’ due to high autofluorescence during flow cytometry experiments [3]. A careful survey of published literature shows that besides the study by Wagner et al. [1], there are a handful of additional publications (Table 1) that have reported scRNAseq on adult mice and human ovaries but all failed to detect the stem cells. Evidently scRNAseq falls short of detecting rare population of stem cells as indicated in the Table 1.

**Table 1:** List of scRNAseq studies on adult mice and human ovaries

**Authors and study details**	**Salient findings**
Fan et al. 2019 Nature Communications [4] Human ovaries	scRNAseq studies on human adult ovaries to develop molecular signature of growing and regressing follicular populations. Study identified different types of granulosa and theca cells, mixture of adaptive and innate immune cells, endothelial and smooth muscle cells. All cell processing was done on * cells collected by centrifuging at 160 g*
Wagner et al. 2020 Nature Communications [1] Human ovaries	Single-cell transcriptomes and cell surface antigen profiles of over 24,000 cells from ovarian cortex samples from 21 patients. Identified transcriptional profiles of six main cell types; oocytes, granulosa cells, immune cells, endothelial cells, perivascular cells, and stromal cells. Adult human ovarian cortex consists of six main cell types and do not harbor germline stem cells *Cells collected by centrifuging at 300 g*
Wang et al. 2020 Cell [5] Monkey ovaries	scRNAseq studies on young and aged ovaries of monkeys identified stromal cells, smooth muscle cells, granulosa cells, endothelial cells, NK cells, macrophages and oocytes with distinct gene-expression signatures Aging was associated with the disturbance of antioxidant signaling specific to early-stage oocytes and granulosa cells. Oxidative damage emerged as a crucial factor in ovarian functional decline with age. Also, inactivated antioxidative pathways, increased reactive oxygen species, and apoptosis were observed in granulosa cells from aged women *Cells collected by centrifuging at 1000 rpm*
Morris et al. 2022 eLife [6] https://doi.org/10.1101/2022.02.08.479522 Mouse ovaries	scRNAseq was used to evaluate the transcriptome of > 34,000 cells of the adult mouse ovary and describe transcriptional changes across estrus cycle and other reproductive states. Major clusters included granulosa cells, stromal cells, endothelial cells, ovary surface epithelial cells, immune cells and oocytes Dynamic mRNA expression in murine ovaries is reported across the estrus cycle and other reproductive states. Extends our understanding of the diversity of cell types in the adult ovary *Speed used for centrifuging cells not mentioned*
Wang et al. 2022 Research Square [7] 10.21203/rs.3.rs-1624864/v1 Human ovaries	Eight different cell types were detected by scRNAseq studies on human ovaries of different ages including granulosa cells, theca and stromal cells, smooth muscle cells, endothelial cells, monocytes, Natural Killer cells, T lymphocytes scRNAseq results picked up cellular senescence and inflammatory changes in aged ovaries. DNA damage genes were upregulated while DNA repair genes were downregulated in the oocytes *Cells collected by centrifuging at 1200 rpm*
Shikanov lab https://event.on24.com/wcc/r/3857131/C1C9EE36B2D21B71D4E49A951201C341?partnerref=WEB Human ovaries	Single cell sequencing and spatial transcriptomics to create a tissue and cell atlas of the human ovary. Analysis of 21,198 cells uncovered four primary cell types found in the human ovary: immune cells, endothelial cells, pericytes, and stromal cells *Speed used for centrifuging cells not mentioned*

This inability of scRNAseq to detect stem cells is not restricted to only the ovaries but stem cells have remained elusive in multiple adult tissues during scRNAseq analysis and the underlying reasons for the same have been explained earlier [8]. Besides being a scarce population, the stem cells pellet down only when centrifuged at 1000 g and as a result they get inadvertently discarded and never get subjected to scRNAseq experiments [8]. This needs to be acknowledged since it is leading to confusions and mis-concepts in the field. It is important to focus on the ovarian (stem cells) biology and let it not be overpowered by technology.

**Fig. 1:**
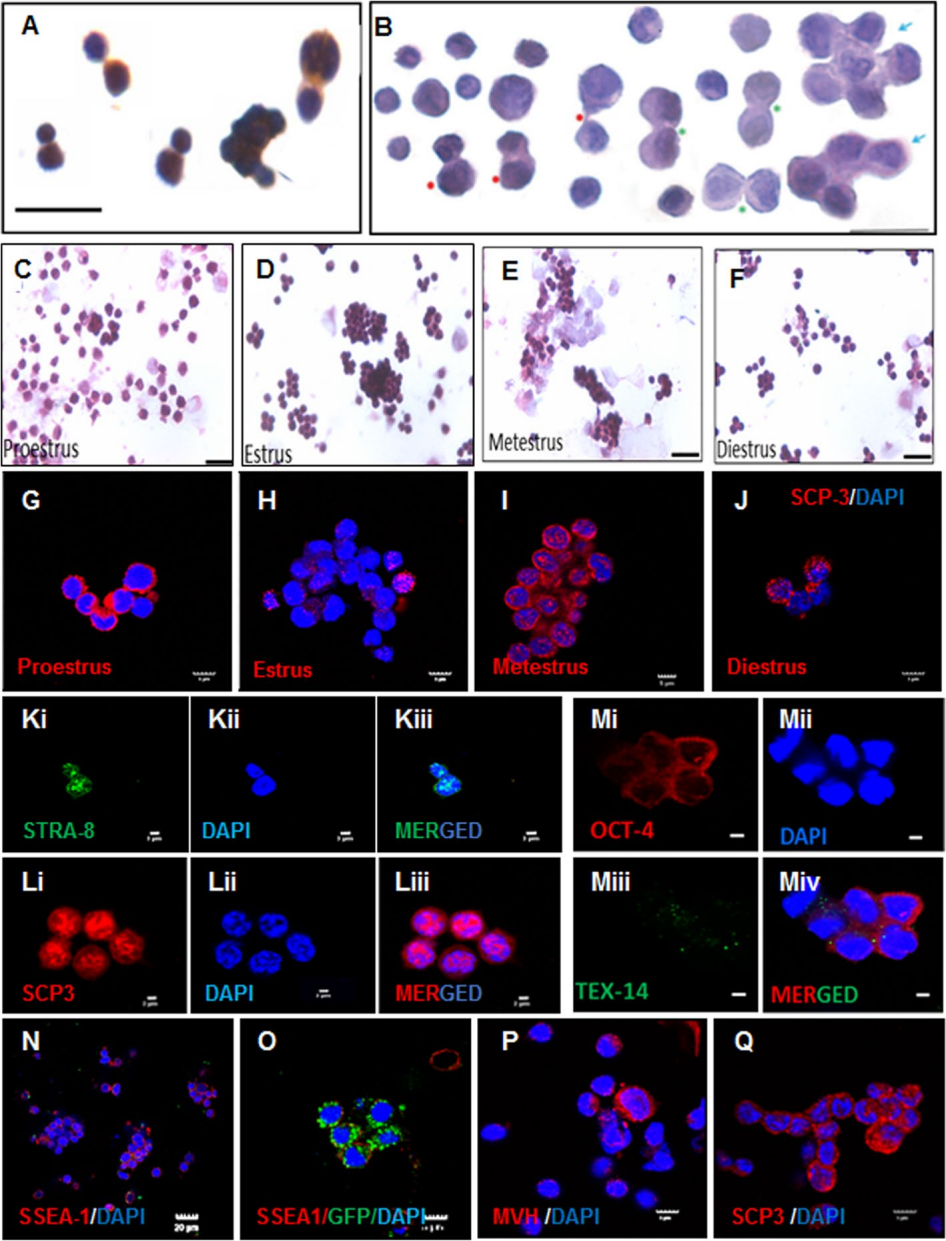
Stem cell kinetics in adult ovaries and uterus. **A** Sheep ovarian stem cells isolated from the ovary surface epithelium are of two distinct sizes and show asymmetrical and symmetrical cell divisions and clonal expansion [14, 15]. These cells were immuno-stained for FSHR. **B** Two populations of stem cells were clearly visualized in the mouse uterus at higher magnification. Asymmetrical (red arrow), symmetrical (green arrow) and clonal expansion showing distinct cytoplasmic connectivity [14]. **C**-**F** Hematoxylin and Eosin stained ovary surface epithelial cells smears obtained from adult mice ovaries during different stages of estrus cycle, showing stem cells and germ cell nest (GCN) formed by clonal expansion across estrus cycle. **G**-**J** Meiotic entry marker synaptonemal complex protein 3 (SCP-3) expression in the GCN during different stages of estrus cycle. It is distinctly cytoplasmic during proestrus suggestive of an oogonial nest, punctate nuclear expression suggests leptotene stage during estrus while the typical crossover pattern during metestrus is suggestive of pachytene stage suggesting gradual maturation and meiosis in the GCN is stage specific. **Ki-iii** Nuclear stimulated by retinoic acid gene 8 (STRA-8) is a specific marker for pre-meiotic germ cells. SCP-3 and STRA-8 expression provide strong evidence to support neo-oogenesis in adult ovary [16]. **Li-iii** SCP-3 expression in DAPI stained nuclei of cells comprising a GCN. **Mi-iv** Co-localization of cytoplasmic OCT-4 and TEX-14 positive ring canals in an oogonial stage GCN. (**N–O**) GCN detected in the OSE isolated from the recipient mice upon transplantation of FACS sorted SSEA-1 + VSELs from a GFP mice. GCN developed from endogenous, tissue-resident VSELs formed SSEA-1 + GCN while the transplanted VSELs formed GFP + SSEA-1 + GCN. Note the GCN were exclusively either GFP-ve or GFP + suggesting their clonal origin [18]. If these were formed by mere aggregation of cells as commented [3], they would show a heterogenous pattern of GFP expression. **P**-**Q** Stem cells and GCN detected in 24 months old mice ovaries showing expression of mouse vasa homolog (MVH) (**P**) and SCP-3 (**Q**). These GCN in aged ovaries are blocked in development into oocytes due to age-related compromised niche as discussed [19]. Both neo-oogenesis in adult life and senescence in aged ovaries have a stem cell basis and VSELs are greatly increased in ovarian cancer [16, 19–21]

In the supplementary section, the authors [3] were critical regarding the presence and biological relevance of very small embryonic-like stem cells (VSELs) in adult ovaries. Adult ovaries harbor two populations of stem cells including VSELs and OSCs [9, 10]. Presence of VSELs was recently confirmed by flow cytometry in mice ovaries by Tilly’s group as well [11]. Being pluripotent, VSELs express nuclear OCT-4 whereas tissue-committed, ‘progenitors’ express cytoplasmic OCT-4 in several adult tissues including ovaries based on both gene transcription and protein expression studies. Transcriptomic data on VSELs isolated from bone marrow and ovaries suggesting classic pluripotent signature and developmental link to primordial germ cells is published [12, 13]. VSELs are very small in size, relatively quiescent and immortal in nature since they undergo asymmetrical divisions to self-renew and give rise to slightly bigger progenitors which are more abundant and undergo symmetrical cell divisions and clonal expansion followed by differentiation into tissue-specific cell types [14]. Clonal expansion of stem cells isolated from sheep and mouse OSE gives rise to germ cell nests (GCN) in response to FSH treatment in vitro [15]. GCNs are not mere aggregates of cells but form due to rapid proliferation and incomplete cytokinesis, are interconnected by TEX-14 positive ring canals and have Cytochrome C and TRAL positive Balbiani body (Fig. 1) [16] similar to as described in fetal ovaries [17]. If they were simple cell aggregates as suggested [3], they should get separated as single cells upon trypsin treatment but rather they are formed by incomplete cytokinesis and show cytoplasmic connectivity (Fig. 1A, B).

Evidence to delineate a definitive role of VSELs in ovarian biology was provided by a transplantation experiment. Upon transplanting, FACS sorted 2–6 µm, GFP + and SSEA-1 + VSELs from FvB-GFP mice into the ovaries of wild-type Swiss mice showed asymmetrical and symmetrical cell divisions resulting in OSCs which further underwent clonal expansion into GFP + GCN [16, 18]. Initial sorted cells were a pure population of VSELs since OSCs being slightly bigger (> 6 µm) in size and with cytoplasmic SSEA-1 (VSELs being pluripotent express cell surface SSEA-1) will get excluded during FACS sorting. GCNs are not mere aggregates of cells but have clonal origin with incomplete cytokinesis was further proved by the observation that all cells in GCNs were either green or non-green (Fig. 1N-O). The fascinating biology of stem cells that reside in the OSE gets altogether missed during scRNAseq studies (Table 1). Morris et al. in 2022 [6] describe OSE as a simple mesothelial cell layer, covering the ovarian stroma, that gets disrupted during ovulation. But OSE is much more dynamic as it houses the stem cells that result in regular GCNs formation, meiosis, neo-oogenesis and primordial follicle assembly during adult life [17].

Instead of enzymatically digesting tissue biopsy and using cells suspension for scRNAseq, another advance is spatial transcriptomic analysis.  This method avoids centrifugation of cells and rather area of interest from the tissue sections are directly used for spatial transcriptomic analysis. Russ et al. [22] carried out spatial transcriptomic profiling of young and aged mice ovaries but still failed to detect stem cells.  They used 8 µm sections and possibly due to the scarce nature of stem cells, the stem cells remained elusive in the study. Sheng et al. [23] carried out both temporal and spatial dynamics of mouse ovaries.  They reported four types of stromal cells and their role in follicle development but stem cells were not detected. Mareckova et al. [24] have reviewed the impact of single-cell genomics on human reproduction towards building cellular atlases which could help to better understand reproductive pathologies but role of stem cells is not highlighted. Our studies on mouse ovarian stem cells (VSELs and OSCs) isolated in the OSE by enzymatic digestion successfully delineated how stem cells function in a subtle manner to undergo neo-oogenesis in adult ovaries [16] and it is stem cells dysfunction that results in senescence of aged ovaries [19] and pathologies like PCOS and POI [25] challenge the data being generated by both temporal and spatial scRNAseq.

Ovarian aging is a complex process resulting in senescence in mice and menopause in women and a topic of intense debate and several possible underlying mechanisms are discussed [26, 27]. It eventually results in the aging of multiple organs and menopausal women suffer from a number of diseases like osteoporosis, cardiovascular disease, obesity, tumors, Alzheimer's disease and diabetes. As a result, ovarian aging has become a key health problem among women and underlying pathomechanisms need to be understood in order to develop methods to delay it especially for women who delay parenthood due to career etc. We have studied ovarian stem cells residing in the OSE in aged ovaries. Ovarian stem cells and GCNs show distinct changes across estrus cycle in adult mice ovaries (Fig. 1) and were observed in large numbers, arrested in meiosis in aged ovaries [19]. The process of neo-oogenesis from the stem cells gets affected with age and ovarian senescence in rodents and menopause in women possibly occurs due to stem cell dysfunction rather than the currently held view of a sudden demise of ovarian follicles resulting in menopause. It has also been reported that Laron-Dwarf and Ames-Dwarf mice with increased numbers of VSELs in adult bone marrow (and most likely also in ovaries) remain fertile up to 2.5 years suggesting that ovarian function depends on VSELs numbers and their normal functioning [28]. scRNAseq studies have been undertaken to understand aging of monkey and human ovaries. Wang et al. [5] reported aging in monkey ovaries is associated with oxidative damage in oocytes and granulosa cells. Wang et al. [7] also used scRNAseq studies to understand age-related changes in human ovaries. They detected several cellular senescence and inflammatory changes in aged ovaries and identified FOXP1 as a central protective factor for ovarian aging. Altogether, these results suggest that scRNAseq failed to throw light on underlying pathomechanisms that result in ovarian aging and menopause which is indeed a stem cell disease as reported by our group [19].

VSELs and OSCs express ERα, ERβ and FSHR [16] and thus are directly vulnerable to endocrine disruption. Rather than genetic changes/mutations, stem cells dysfunctions due to endocrine disrupting chemicals result in defective oogenesis observed in PCOS and POI ovaries in a recently published study on mice [25] and these endocrine disrupting chemicals also affect aging [29]. VSELs are also implicated in ovarian cancers [20, 21]. ScRNAseq has failed to detect these stem cells in cancer tissues as well [30, 31]. Vuong et al. [30] carried out a study on mice OSE cells exposed to estradiol, failed to detect stem cells and concluded that estradiol increases epithelial cells susceptibility to tumor initiation. A careful look at their method of isolation of OSE cells for scRNAseq showed that they centrifuged cells at 1000 rpm which resulted in the loss of stem cells. Epithelial cells do not initiate ovarian cancer, rather it is the pluripotent VSELs residing amongst them that initiate cancer [20, 21, 32]. Cancer is indeed a stem cell disease and both PCOS and POI also occur due to stem cells dysfunction.

To conclude, a careful relook at the protocols for sc-RNAseq is required and a novel role of stem cells has emerged in regulating both normal ovarian biology and pathology which needs to be confirmed by other groups in the field. This will hopefully provide newer opportunities for managing ovarian pathologies including cancer in future.

## Data Availability

EPI/OTH/02/2022

## Abbreviations

OSCs: Ovarian stem cells
OSE: Ovary surface epithelium
VSELs: Very small embryonic-like stem cells
GCNs: Germ cell nests
PCOS: Polycystic ovarian syndrome
POI: Premature ovarian insufficiency
