# Correction: VSELs and OSCs together sustain oogenesis in adult ovaries and their dysfunction results in age-related senescence, PCOS, POI and cancer

**DOI:** 10.1186/s13048-023-01255-6

**Published:** 2023-08-14

**Authors:** Deepa Bhartiya, Diksha Sharma

**Affiliations:** 1 Stem Cell Biology Department, ICMR-National Institute for Research in Reproductive & Child Health, Jehangir Merwanji Street, Parel, Mumbai, 400 012 India; 2 Present Address: Epigeneres Biotech Pvt Ltd, Sun Mill Compound, Senapati Bapat Marg, Lower Parel, Mumbai, 400 013 India

**Correction: J Ovarian Res 16:29 (2023)**

10.1186/s13048-022-01093-y

Following publication of the original article [1], please note that Fig. 1A-F are composites prepared by putting together various fields as these cells are spread far apart on the slides.
